# Diagnostic Challenges in Pediatric Urology: A Case Report and Literature Review on Hourglass Bladder

**DOI:** 10.7759/cureus.47772

**Published:** 2023-10-26

**Authors:** Francesco F Comisi, Elena Esposito, Valeria Manca, Giuseppe Masnata

**Affiliations:** 1 Pediatrics, University of Cagliari, Cagliari, ITA; 2 Pediatric Urology and Urodynamics, Brotzu Hospital, Cagliari, ITA

**Keywords:** ultrasound, scintigraphy, bladder diverticulum, congenital urinary anomalies, hourglass bladder

## Abstract

Hourglass bladder is a definition used to describe a particular configuration of the urinary bladder, divided into two compartments, upper and lower, communicating through a narrowed segment resembling an hourglass. It may be due to various conditions, such as bladder diverticula, bladder neck obstruction, neurogenic bladder, or other abnormalities. Congenital hourglass bladder is an extremely rare anomaly. To the best of our knowledge, only 24 cases have been reported. We present the case of a 2-year-old male, probably the youngest patient with congenital hourglass bladder ever recorded. We aim to increase knowledge about the incidence of this likely underdiagnosed condition and its management and stress the importance of long-term follow-up.

## Introduction

True hourglass bladder is a congenital malformation, most common in males, caused by a partial transverse septum that divides the cavity into an upper and a lower compartment [1,2]. The communicating aperture varies in diameter from 1 to 5 cm. The relative size of the upper and the lower compartments is variable. The ureteral orifices may open into the upper compartment or in the lower one [1]. The etiological factor responsible in the embryo for the constriction which divides the bladder into two cavities is not known, but it may be due to an atavistic relationship to the hourglass bladder normally found in some animals, the persistence of the ureteric membrane, or unequal growth [2]. This condition may be diagnosed as an incidental finding or manifest following the onset of urinary complications, like incomplete bladder voiding, cystitis, dysuria, or other lower urinary tract symptoms. In the last two decades, the medical literature has noted only a single instance of congenital hourglass bladder [1], while several cases have been reported as postoperative complications of bladder reconstructive surgery or in association with other abnormalities of the urinary tract. We report the case of a 2-year-old male with a congenital hourglass bladder.

## Case presentation

**Figure 1:**
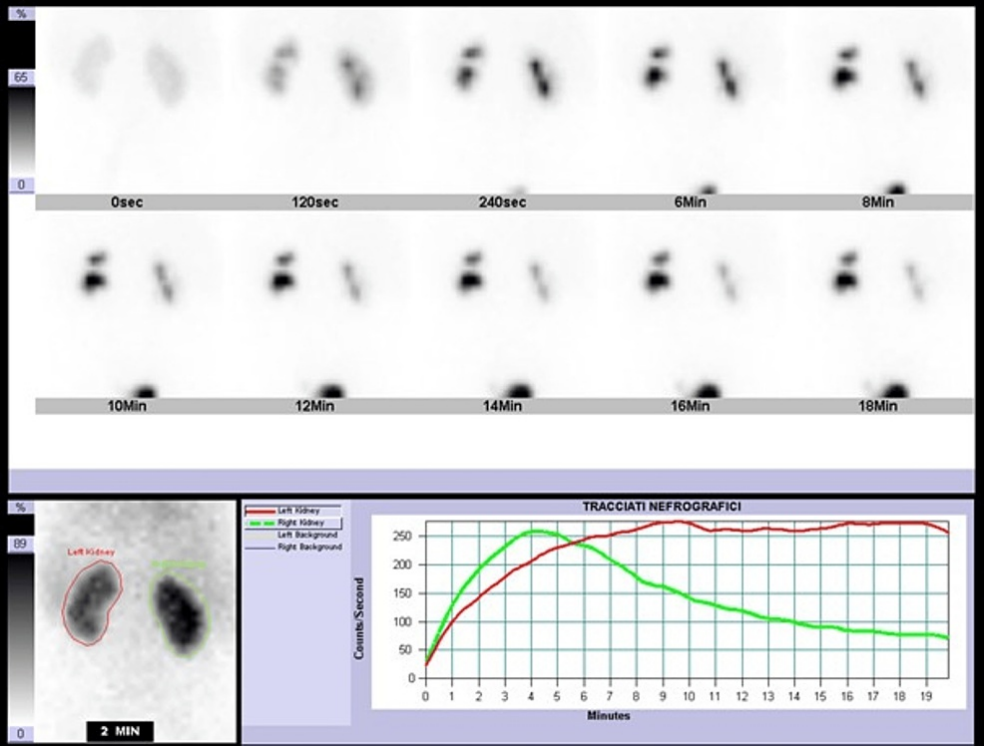
99mTc-MAG3 dynamic renal scintigraphy showing normal total tubular excretion

A 2-year-old male was born at 40 weeks of gestation by normal pregnancy and delivery to non-consanguineous parents. His birth weight was 3,270 grams, without complications during the perinatal period. Prenatal ultrasound, performed at 32 weeks of gestation, revealed a 13 mm left renal calyceal-pelvic dilation, reduced to 10 mm by the 35th gestational week. When the patient was two weeks old, an abdominal ultrasound detected mild ectasia of the renal calyces and a minor dilatation of the extrarenal pelvis. Subsequently, micturating cystourethrography (MCU) showed fourth-grade, left vesicoureteral reflux with an unclear focal dilation, initially interpreted as either enlargement of the terminal ureter or a diverticulum. Antibiotic prophylaxis was started. His physical examination was unremarkable. He had no difficulty with micturition and never experienced UTIs. Renal function testing revealed normal serum urea, creatinine, and electrolyte levels. The cytology of the urine specimen was normal. Technetium-99m mercaptoacetyltriglycine (99mTc-MAG3) dynamic renal scintigraphy further confirmed normal kidney function, with an 83.3 ml/min tubular excretion rate, ruling out any obstructive forms (Figure 1).

**Figure 2:**
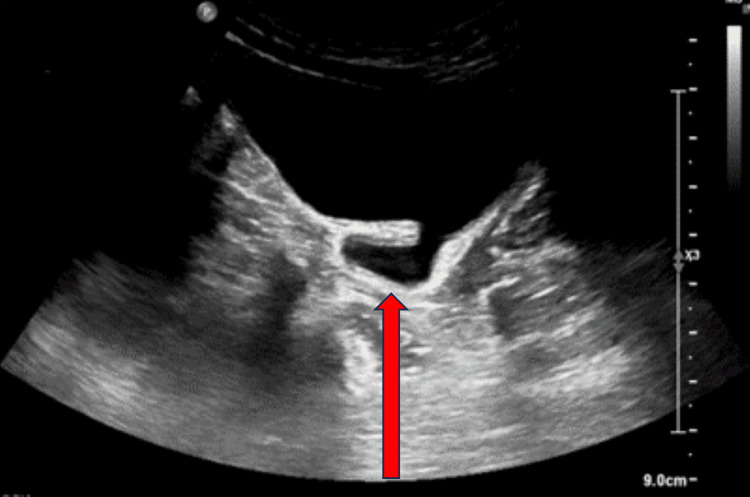
Ultrasound image of the urinary bladder showing the two compartments of the hourglass urinary bladder

**Figure 3:**
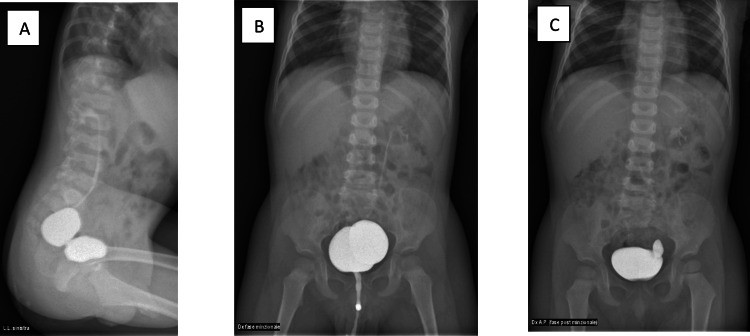
MCU images (A) Upper and lower compartments of the hourglass urinary bladder. (B) Voiding phase. (C) Post voiding phase

At the age of two years, urinary ultrasonography (Figure 2) and MCU (Figure 3) were repeated, showing an hourglass bladder with second-grade left vesicoureteral reflux and ectopic ureter.

## Discussion

Hourglass bladder may occur in patients with mixed neurogenic urinary incontinence or, more commonly, in children following seromuscular colocystoplasty and patients undergoing bladder auto-augmentation to treat neuropathic bladder secondary to myelomeningocele [3-5]. In 2009, Vaidyanathan et al. described a patient with an hourglass bladder as a late complication of suprapubic cystostomy [6]. In 2015, Alhazmi reported a case occurring after ileocystoplasty [7]. Congenital hourglass bladder is an extremely rare anomaly, warranting consideration in the differential diagnosis with conditions such as bladder duplication, bladder diverticula, and other urological malformations. The diagnosis is usually made by cystography and endoscopy. CT and MRI are also possible options, as Matsumoto et al. reported in 1995 [8]. According to Ockerblad et al., most patients manifest urinary disturbance since early life, including urinary difficulty, dysuria, or enuresis, while a minority have no symptoms until later in life. The development of severe cystitis is probably responsible for most of the symptoms [2]. That is the case of a patient presented by Zellermayer et al. in 1944, who gave a history of lifelong bladder disturbances, including dysuria, enuresis, and cystitis [9]. Severe manifestations, such as acute urinary retention and haematuria, are reported, too [9]. The main aim of this paper is to highlight the importance of follow-up in case of early detected anomalies to prevent major complications.

## Conclusions

The reported patient’s first MCU was unclear; dilation of the terminal ureter and bladder diverticulum were possible diagnoses. Adherence to a strict clinical and radiological follow-up, with periodic ultrasounds and repeated MCUs, allowed us to detect his rare congenital deformity and assess the evolution of the clinical scenario from a very young age.

This case underscores the importance of a multidisciplinary approach involving different imaging modalities. Long-term follow-up for such cases is necessary. It should include urinalysis, abdominal ultrasonography, and a daily voiding diary to detect urinary tract infections early and micturition complications affecting the voiding or post-voiding phase. MCU and urodynamic testing may be repeated if the ultrasound reveals structural anomalies in the urinary tract.
